# Inhibition of lysine‐specific demethylase 1A suppresses neointimal hyperplasia by targeting bone morphogenetic protein 2 and mediating vascular smooth muscle cell phenotype

**DOI:** 10.1111/cpr.12711

**Published:** 2019-11-18

**Authors:** Xiaobo Zhang, Tao Huang, Heng Zhai, Wenpeng Peng, Yong Zhou, Qi Li, Haifeng Yang

**Affiliations:** ^1^ Department of Cardiology Union Hospital Tongji Medical College Huazhong University of Science and Technology Wuhan China; ^2^ Department of Neurosurgery Union Hospital Tongji Medical College Huazhong University of Science and Technology Wuhan China; ^3^ Department of Neurology Union Hospital Tongji Medical College Huazhong University of Science and Technology Wuhan China

**Keywords:** bone morphogenetic protein 2, (K)‐specific demethylase 1A, phenotype, vascular disease, vascular smooth muscle cells

## Abstract

**Objectives:**

Vascular disorders are associated with phenotypical switching of vascular smooth muscle cells (VSMCs). We investigated the effect of bone morphogenetic protein (BMP)‐2 in controlling VSMC phenotype and vascular disorder progression. Lysine (K)‐specific demethylase 1A (KDM1A) has been identified to target BMP‐2 and is employed as a therapeutic means of regulating BMP‐2 expression in VSMCs.

**Materials and methods:**

VSMCs were stimulated with angiotensin II, and the expression of KDM1A and BMP‐2 was detected. VSMC proliferation, apoptosis, and phenotype were evaluated. An in vivo aortic injury model was established, and VSMC behaviour was evaluated by the expression of key markers. The activation of BMP‐2–associated signalling pathways was examined.

**Results:**

We confirmed the inhibitory effect of KDM1A on BMP‐2 activity and demonstrated that KDM1A inhibition prevented VSMC transformation from a contractile to synthetic phenotype. In angiotensin II‐treated VSMCs, KDM1A inhibition triggered a decrease in cell proliferation and inflammatory response. In vivo, KDM1A inhibition alleviated post‐surgery neointimal formation and collagen deposition, preventing VSMCs from switching into a synthetic phenotype and suppressing disease onset. These processes were mediated by BMP‐2 through canonical small mothers against decapentaplegic signalling, which was associated with the activation of BMP receptors 1A and 1B.

**Conclusions:**

The regulatory correlation between KDM1A and BMP‐2 offers insights into vascular remodelling and VSMC phenotypic modulation. The reported findings contribute to the development of innovative strategies against vascular disorders.

## 1 INTRODUCTION

Neointimal hyperplasia is a pathological process that often occurs after surgical intervention in vascular diseases such as pulmonary arterial hypertension and is the main contributor to post‐intervention restenosis.^1^ It is characterized by the excess proliferation and migration of vascular smooth muscle cells (VSMCs), promoting the development of cardiovascular diseases with cytopathological manifestations such as vascular stenosis and calcification.^2^, ^3^, ^4^ VSMCs exist in and can interconvert between two phenotypic states, namely the contractile (differentiation) and synthetic (dedifferentiation) states.^4^, ^5^ The transformation from a contractile state to a synthetic one leads to enhanced VSMC proliferation and migration, secretion and synthesis of extracellular matrices, and formation of neointimal membranes, which is the key step in the initiation of severe vascular proliferative diseases.^6^, ^7^ Precise control of the phenotypic transformation of VSMCs is thus an important step in preventing the occurrence and development of the above‐mentioned diseases at an early stage.

The phenotypic transformation of VSMCs is mainly induced by growth factors (eg, angiotensin II), mechanical stimulation, and molecular signalling. The stimulation signal is transmitted to the nucleus and eventually regulates the expression of smooth muscle cell differentiation marker genes.^8^, ^9^, ^10^ Among the members of the bone morphogenetic protein (BMP) multifunctional cytokine family, BMP‐2 is a well‐known osteogenic factor^11^, ^12^ that also plays critical roles in embryonic development,^13^, ^14^ nerve growth,^15^, ^16^ and cell behaviour regulation.^17^ BMP‐2 signalling can occur via either canonical or non‐canonical routes. Canonical BMP‐2 signalling is closely linked to downstream small mothers against decapentaplegic (SMAD) signalling, involving BMP‐transduced phosphorylation of receptor‐regulated (R)‐SMADs 1, 5, and 8 via the activation of BMP‐2 receptors (BMPRs).^18^ On the other hand, non‐canonical BMP signalling triggers non‐SMAD activity including mitogen‐activated protein kinase and phosphoinositide 3 kinase pathways.^18^, ^19^ In tumour‐related studies, BMP‐2 has demonstrated an inhibitory effect on colon cancer cell growth and proliferation while promoting apoptosis.^20^, ^21^ In addition, BMP‐2 signalling is involved in the maintenance of the contractile phenotype and has been shown to inhibit VSMC proliferation and neointimal hyperplasia.^22^, ^23^ Whether BMP‐2 signalling is mediated by canonical or non‐canonical routes in vascular remodelling remains to be investigated.

We hypothesized that BMP‐2 signalling is directly or indirectly involved in the phenotypic transformation of VSMCs and that the occurrence of vascular diseases may be prevented by regulating BMP‐2 expression. Transcriptome sequencing has identified lysine‐specific histone demethylase 1 (or lysine (K)‐specific demethylase 1A, KDM1A) as a compound that potentially targets BMP‐2,^24^ suggesting that its function in cardiovascular health may be non‐negligible. Studies on KDM1A have revealed that histone methylation occurs through the interaction of histone methyltransferase and demethylase, dynamically regulating biological processes such as the activation and inhibition of gene transcription.^25^ Presently, research on KDM1A has focused on tumours, and it has been found to be highly expressed in tumour tissues and cells, participating in multiple signalling pathways that affect tumorigenesis, tumour invasion and metastasis, and drug resistance.^26^ For example, high levels of KDM1A are often closely associated with the clinical progression of highly aggressive medulloblastoma, whereas inhibition of KDM1A using drugs reduced the tumorigenicity of human neuroblastoma cell lines in nude mice.^24^ Yet, the involvement of KDM1A in the phenotypic transformation of VSMCs, the occurrence of neointimal hyperplasia and the development of vascular diseases have not been investigated.

Herein, we induced phenotypic switching of VSMCs using angiotensin II and investigated the resulting changes in KDM1A and BMP‐2 expression. Cell behaviour including proliferation, migration, and apoptosis and the secretion of a variety of related factors were examined to validate the conversion of VSMCs from a contractile to a synthetic state. A rat model of aortic endothelial balloon injury was established to further investigate the relationship between KDM1A and BMP‐2 at the in vivo level and to elucidate the possible involvement of BMPRs and SMADs in the phenotypic remodelling of VSMCs. The findings of our study aim to clarify the mechanism of KDM1A in regulating the biological behaviour of VSMCs and neointimal formation, revealing new targets and insights for the prevention and treatment of vascular proliferative diseases.

## 2 MATERIALS AND METHODS

Detailed Materials and Methods can be found in the online Materials S1.

### 2.1 VSMC isolation, culture and treatment

Vascular smooth muscle cells were extracted from the thoracic and abdominal aortic tissues of Sprague Dawley rats following standard procedures for isolating primary cells. Cells were cultured with smooth muscle culture medium until they reached 80%‐90% confluence. Morphological characterization via bright‐field and fluorescence microscopy was carried out to confirm the successful extraction of VSMCs, which typically show peak‐valley structures. To induce phenotypic switching of VSMCs, the cells were treated with angiotensin II (Ang‐II) with or without pre‐treatment using ORY‐1001, a specific inhibitor of KDM1A (denoted as KDM‐inh). Cells that were treated with neither Ang‐II nor KDM‐inh were used as controls.

### 2.2 Evaluation of VSMC proliferation and apoptosis

Vascular smooth muscle cells were treated as described previously and subjected to characterization of proliferation and apoptosis. The viability of Ang‐II‐induced VSMCs was first assessed using a 3‐(4,5‐dimethylthiazol‐2‐yl)‐2,5‐diphenyltetrazolium bromide (MTT) assay based on the instructions provided in the assay kit. Then, VSMC proliferation was evaluated using a scratch assay. Images of scratches in the cell monolayer were acquired immediately or 24 hours after scratching, and the wound‐healing rate was calculated using imagej. EdU staining was also carried out to visualize VSMC proliferation following the instructions provided in the EdU staining kit. The percentage of EdU‐positive cells was calculated using ImageJ. To examine the migratory ability of treated or non‐treated VSMCs, a Transwell migration assay was performed following the manufacturer's instructions. Cell cycle progression and apoptosis were analysed using flow cytometry.

### 2.3 Identification of VSMC phenotype

The phenotype of VSMCs was identified by immunofluorescence staining of α‐smooth muscle actin (α‐SMA) and osteopontin (OPN), which are markers of the contractile and synthetic phenotypes, respectively. After treatment as described previously, the VSMCs were fixed, permeabilized, blocked, and incubated with respective primary antibodies overnight at 4°C. After incubation with the corresponding secondary antibodies for 1 hour at 37°C, the cells were observed using a fluorescence microscope. The production of growth factors and inflammatory cytokines, which also function as markers of VSMC phenotype, was assessed using enzyme‐linked immunosorbent assay kits following the manufacturer's instructions. The following growth factors and cytokines were evaluated: matrix metalloproteinase‐2 (MMP‐2), intercellular adhesion molecule‐1 (ICAM‐1), platelet‐derived growth factor (PDGF), fibroblast growth factor‐2 (FGF‐2), interleukin (IL)‐6, IL‐18, monocyte chemotactic protein‐1 (MCP‐1), and transforming growth factor‐β (TGF‐β).

### 2.4 mRNA and protein expression of key markers

Quantitative reverse transcription‐polymerase chain reaction was performed to detect the mRNA expression of KDM1A, and BMP‐2 after VSMCs was treated as described previously, using GAPDH as a housekeeping gene. The primer sequences were as follows: KDM1A forward, 5′‐GCTCCTATTCTTATGTGG‐3′ and reverse, 5′‐AGTTGCGGATTGTATG‐3′; BMP‐2 forward, 5′‐GCGAGTTTGAGTTGAGG‐3′ and reverse, 5′‐TGAGCACGGTGTTGG‐3′; GAPDH forward, 5′‐CAAGTTCAACGGCACAG‐3′ and reverse, 5′‐CCAGTAGACTCCACGACAT‐3′. Western blot was carried out to quantify the relative protein expression of KDM1A, BMP‐2, α‐SMA, and OPN. Protein expression was normalized to that of GAPDH. The intensity of the protein bands was acquired using Tanon software.

### 2.5 In vivo rat model of aortic endothelial artery balloon injury

All animal experiments were performed in accordance with the Guidelines for Animal Care and Use of the Model Animal Research Institute at Wuhan Myhalic Biotechnology Co Ltd. and were approved by the institutional review board (approval number: HLK‐20180925‐01). Sprague Dawley rats were subjected to either sham operation (Control) or aortic endothelial balloon injury via surgical dissection of the common carotid artery. The aortic endothelial balloon injury procedure was performed by inserting a balloon catheter into the left common carotid artery down to the abdominal aorta. The balloon was filled with physiological saline, and injury was induced by pulling the catheter to remove the arterial endothelium. Injured rats were treated with KDM‐inh and/or recombinant BMP‐2, and aortic tissues were collected from the rats 7, 14, or 28 days after the operation for further characterization and analysis.

### 2.6 Histological and immunohistochemical evaluation of injured tissues

The collected aortic tissues were cut into pieces, fixed, and dehydrated by conventional means. Tissue specimens were embedded in paraffin blocks and sliced at a thickness of approximately 5 μm. Then, the tissue sections were subjected to haematoxylin and eosin (H&E) staining to observe general tissue morphology and Masson's trichrome staining to evaluate collagen deposition, using well‐established histological protocols. The ratio of intimal to medial (I/M) thickness was calculated using ImageJ, and collagen deposition was quantified using Image‐Pro Plus. The tissues were also subjected to immunohistochemical staining for KDM1A, BMP‐2, α‐SMA, OPN, and proliferating cell nuclear antigen (PCNA). After nuclear staining, dehydration, and mounting, the stained tissue samples were viewed and analysed using a microscope.

### 2.7 Involvement of BMPR and SMAD signalling in neointimal hyperplasia

Proteins were extracted from aortic tissues of rats subjected to aortic endothelial balloon injury and/or treated with KDM‐inh and/or BMP‐2. To investigate the role of BMPR signalling, western blot was performed to quantify the protein expression of BMPR‐1A, BMPR‐1B, and BMPR‐2. Protein expression was normalized to that of GAPDH. To explore the involvement of SMAD signalling pathways, western blot was carried out to examine the phosphorylated levels of individual SMADs (1, 5, and 8) relative to the respective levels of total SMADs. The intensity of the protein bands was acquired using tanon software.

### 2.8 Statistical analysis

All experiments were performed in triplicates (n = 3), and the data are presented as the mean ± standard deviation (SD). Statistical analysis was carried out by one‐way analysis of variance with Tukey's test for multiple comparisons using originpro 8. imagej ver. 1.8 and image‐pro plus 6.0 were used for image analysis. *P* < .05 is considered statistically significant.

## 3 RESULTS

### 3.1 Ang‐II enhanced VSMC proliferation and revealed inverse correlation between KDM1A and BMP‐2

**Figure 1:**
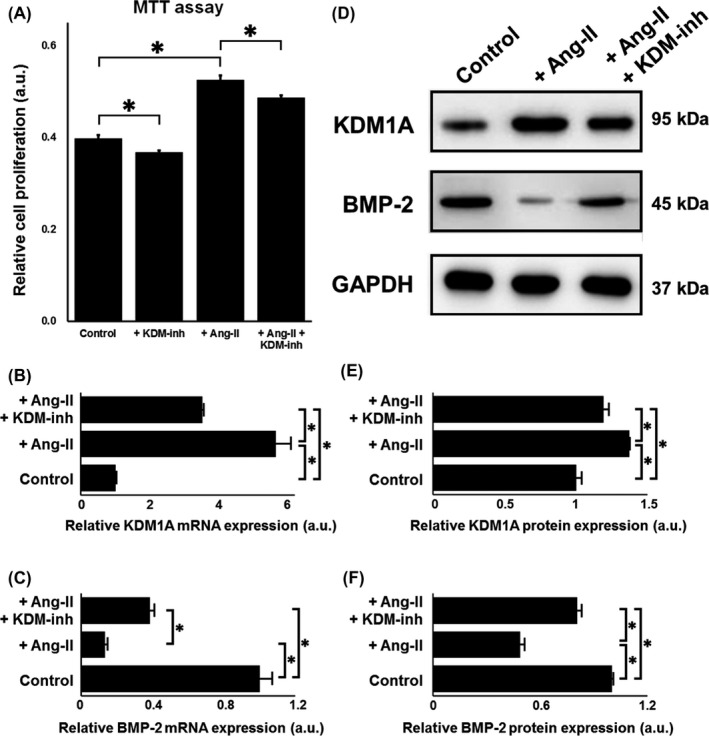
Effect of Ang‐II on VSMC proliferation and the expression of KDM1A and BMP‐2. (A) MTT assay of VSMC proliferation with and without Ang‐II and/or KDM‐inh stimulation. qRT‐PCR of the relative mRNA expression of (B) KDM1A and (C) BMP‐2 (both normalized to GAPDH) in VMSCs with and without Ang‐II stimulation, in the absence or presence of a KDM‐inh. (D) Western blot detection and quantification of the relative protein expression of (E) KDM1A and (F) BMP‐2 in VMSCs with and without Ang‐II stimulation, in the absence or presence of a KDM‐inh. For (D), portions of full Western blot membrane images were cropped to show relevant bands. The data are presented as the mean ± SD of three independent replicates, *P < .05

Isolated VSMCs (Figure S1 in Materials S1) were stimulated with Ang‐II and/or KDM‐inh to induce phenotypical switching from the contractile to synthetic (or proliferative) state. We observed a notable increase in the proliferation of Ang‐II–stimulated VSMCs compared to that of control cells, as revealed by MTT assay (Figure 1A), whereas KDM‐inh suppressed the proliferation of both control and Ang‐II–induced VSMCs. The mRNA expression of KDM1A was upregulated by Ang‐II (*P* < .05; Figure 1B), whereas that of BMP‐2 was downregulated (*P* < .05; Figure 1C), exhibiting an inverse relationship with KDM1A. Meanwhile, the addition of an inhibitor of KDM1A (KDM‐inh) counteracted the effects of Ang‐II. In agreement with mRNA expression, the protein expression of KDM1A (*P* < .05; Figure 1D,E) and BMP‐2 (*P* < .05; Figure 1D,F) showed the same trends as their respective mRNA counterparts, with KDM‐inh playing an antagonistic role against Ang‐II in VSMCs.

**Figure 2:**
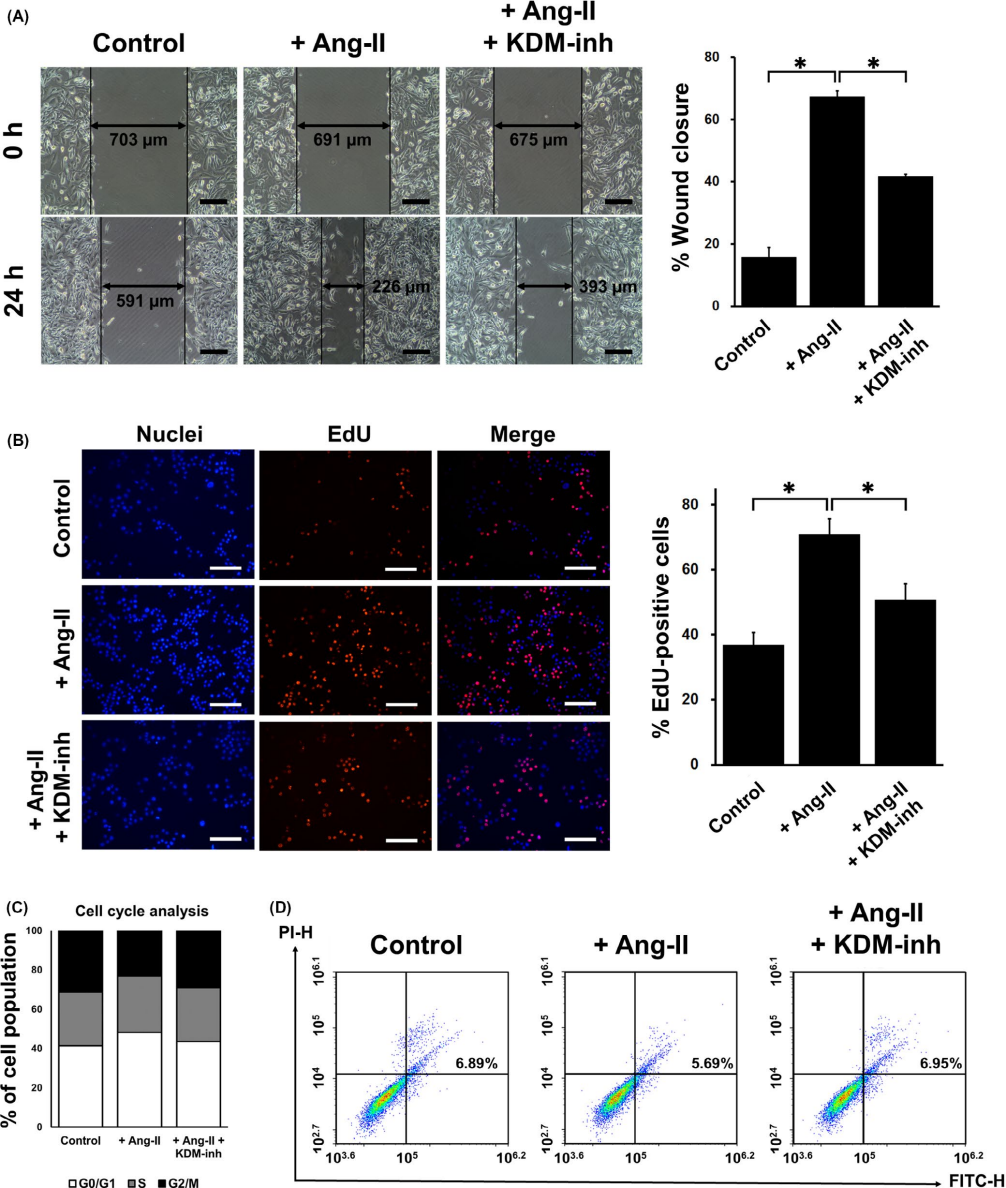
Proliferative and apoptotic behaviour of VMSCs. (A) Scratch assay and quantification of the percentage of wound closure in VMSCs with and without Ang‐II stimulation, in the absence or presence of a KDM‐inh, showing gap closure at the time of scratching (0 h) and 24 h after scratching. Wound healing (%) was calculated using ImageJ. Scale bar, 200 μm. The data are presented as the mean ± SD of three independent replicates, *P < .05. (B) EdU staining and quantification of EdU‐positive proliferating VMSCs with and without Ang‐II stimulation, in the absence or presence of a KDM‐inh. Nuclei are shown in blue (DAPI), and proliferating cells (EdU) are shown in orange. “Merge” indicates the composite of the “Nuclei” and “EdU” images. The percentage of EdU‐positive cells was calculated using ImageJ. Scale bar, 100 μm. The data are presented as the mean ± SD of three independent replicates, *P < .05. Flow cytometric analysis of (C) cell cycle progression and (D) apoptosis

We further examined the impact of Ang‐II on VSMC proliferation (Figure 2A) and migration (Figure S2 in Materials S1) using a scratch assay and Transwell assay, respectively. We noted that 24 hours after scratching, cells stimulated with Ang‐II proliferated with greater capacity than did control cells, closing the scratch gap more than three times more efficiently. However, in the presence of KDM‐inh, the proliferative capacity of the VSMCs was hindered, and wound closure did not proceed as effectively. EdU staining confirmed these results, showing that Ang‐II promoted whereas KDM‐inh suppressed the proliferation of VSMCs (Figure 2B). Additionally, examination of cell cycle progression showed that Ang‐II promoted the entry of VSMCs into the G0/G1 phase, whereas KDM‐inh reduced the proportion of cells in the G0/G1 phase to a level similar to that of control cells (Figure 2C). VSMC apoptosis was correspondingly suppressed by Ang‐II (Figure 2D), though not to a drastic extent. Again, KDM‐inh counteracted the effects of Ang‐II as anticipated.

### 3.2 Ang‐II‐induced contractile‐to‐synthetic transition of VSMCs requires KDM1A

**Figure 3:**
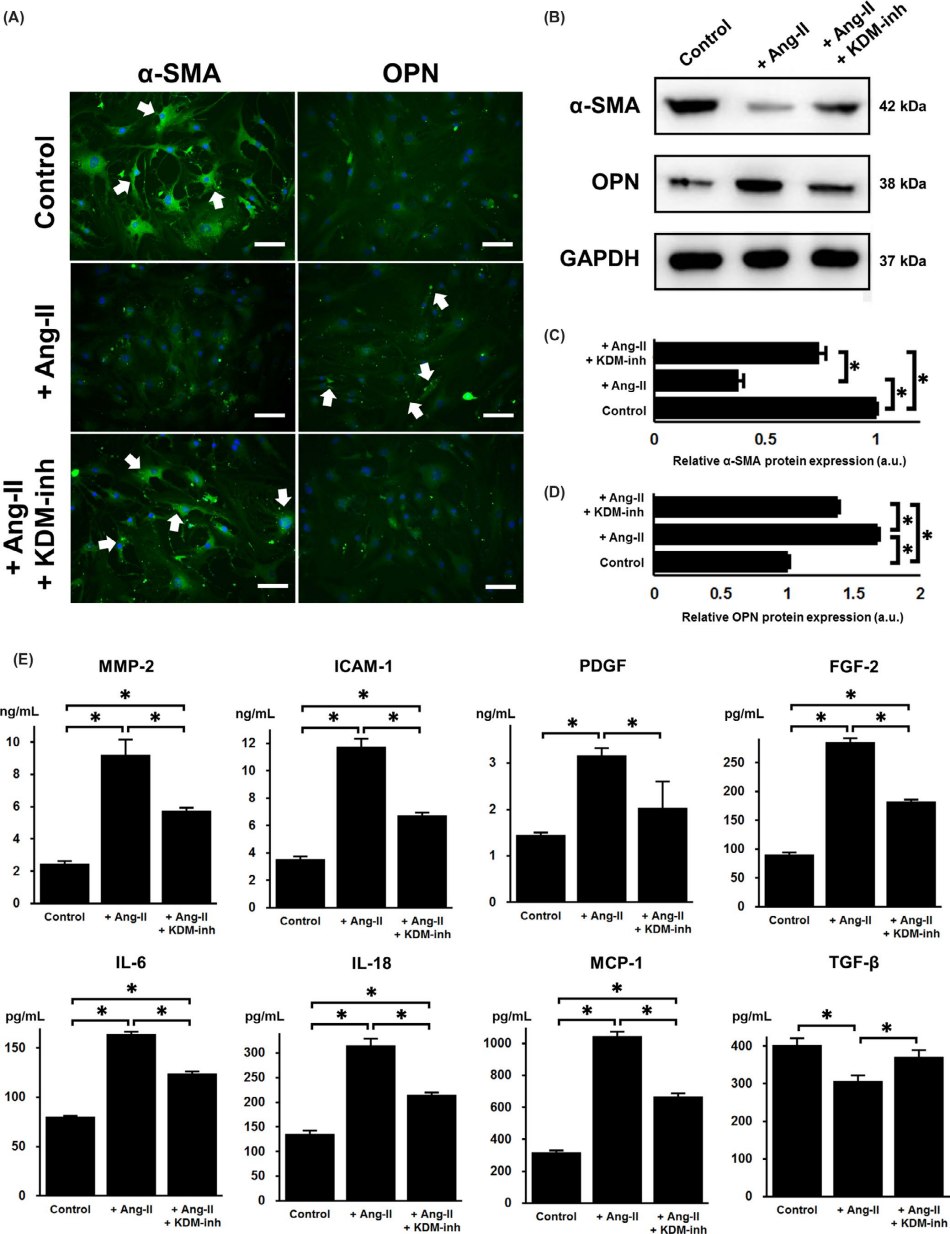
In vitro characterization of contractile‐to‐synthetic transition in VSMCs. (A) Immunofluorescence staining of α‐SMA, a marker of the contractile phenotype, and OPN, a marker of the synthetic/proliferative phenotype, in VMSCs with and without Ang‐II stimulation, in the absence or presence of a KDM‐inh. White arrows point to areas of positive staining (green). Scale bar, 100 μm. (B) Western blot detection and quantification of the relative protein expression of (C) α‐SMA and (D) OPN (both normalized to GAPDH) in VMSCs with and without Ang‐II stimulation, in the absence or presence of a KDM‐inh. (E) ELISA of the secretion of factors involved in phenotypic regulation (MMP‐2, ICAM‐1, PDGF, FGF‐2, IL‐6, IL‐18, MCP‐1, and TGF‐β) in VMSCs with and without Ang‐II stimulation, in the absence or presence of a KDM‐inh. For (B), portions of full western blot membrane images were cropped to show relevant bands. The data are presented as the mean ± SD of three independent replicates, *P < .05

To characterize the phenotypic transformation of VSMCs under Ang‐II stimulation, the cells were subjected to immunofluorescence staining for α‐SMA and OPN (Figure 3A), which are markers of the contractile and synthetic states, respectively. We noted that while VSMCs initially showed high expression of α‐SMA, positive staining was significantly weaker in VSMCs treated with Ang‐II, whereas the KDM‐inh restored the intensity of positive staining in Ang‐II–treated cells. The opposite phenomenon was observed for OPN, as Ang‐II increased its expression in VSMCs while the KDM‐inh caused a decrease in its expression. Western blot of the protein expression of α‐SMA (*P* < .05; Figure 3B,C) and OPN (*P* < .05; Figure 3B,D) was in agreement with the results of immunofluorescence. We further monitored the phenotypic transformation of VSMCs by measuring the secretion of a variety of growth factors and inflammatory cytokines known to be associated with VSMC behaviour (Figure 3E). The expression of PDGF, FGF‐2, and MMP‐2, which are all markers of the synthetic phenotype in VSMCs,^27^, ^28^, ^29^, ^30^ was elevated by Ang‐II (*P* < .05), whereas that of the contractile marker TGF‐β^29^, ^31^ was downregulated (*P* < .05). Additional inflammatory markers of the synthetic state (ICAM‐1, MCP‐1, IL‐6, and IL‐18)^32^, ^33^ also showed a significant increase in expression upon Ang‐II stimulation (*P* < .05). In all cases, the presence of a KDM‐inh counteracted the action of Ang‐II (*P* < .05).

### 3.3 KDM1A and BMP‐2 showed inverse correlation in in vivo aortic injury model

**Figure 4:**
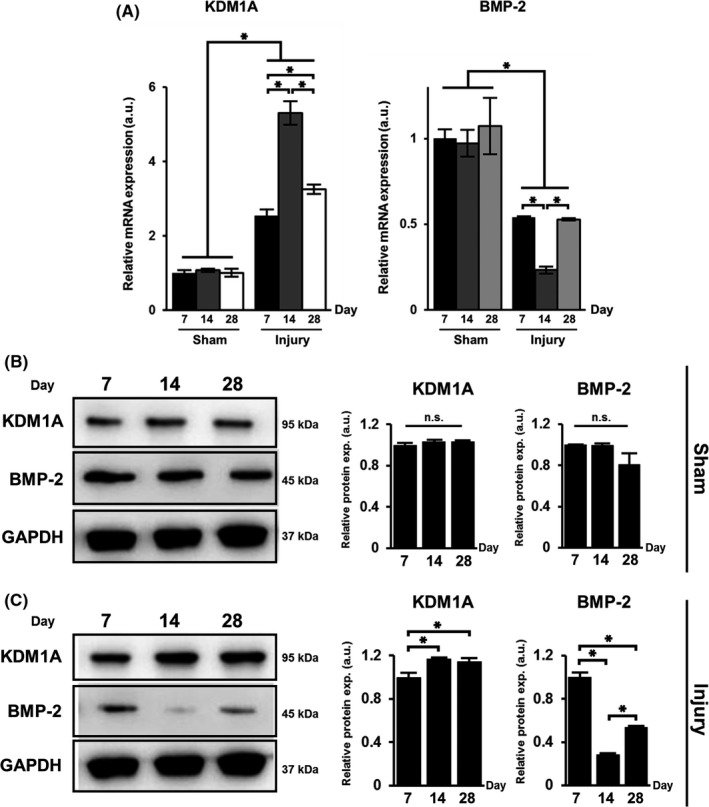
Expression of KDM1A and BMP‐2 in aortic tissues of rats subjected to aortic endothelial balloon injury. (A) qRT‐PCR of the relative mRNA expression of KDM1A and BMP‐2, performed 7, 14, and 28 d after experimental rats were subjected to either sham surgery or balloon injury in the aorta. Western blot detection and quantification of the relative protein expression of KDM1A and BMP‐2 (both normalized to GAPDH), performed 7, 14, and 28 d after experimental rats were subjected to (B) sham surgery or (C) balloon injury in the aorta. For (B) and (C), portions of full western blot membrane images were cropped to show relevant bands. The data are presented as the mean ± SD of three independent replicates, *P < .05; n.s., not significant at *P* < .05

In an in vivo model of aortic endothelial aortic endothelial balloon injury established in rats, we observed that the mRNA expression of KDM1A in the aortic tissues of rats subjected to injury was remarkably elevated on day 7, 14, and 28 post‐surgery, compared to that sham‐operated rats (*P* < .05). On the contrary, the mRNA expression of BMP‐2 showed a significant decline with balloon injury (*P* < .05). The maximum and minimum mRNA expression of KDM1A and BMP‐2, respectively, was detected on day 14 (Figure 4A). Analysis of protein expression showed no differences in the expression of KDM1A and BMP‐2 in sham‐operated rats throughout the observation period of 28 days (Figure 4B). However, in rats subjected to balloon injury, the protein expression of KDM1A was upregulated 14 and 28 days post‐surgery compared to that on day 7 (*P* < .05). BMP‐2 expression was downregulated on days 14 and 28 compared to that on day 7 (*P* < .05), corresponding to the trend of mRNA expression (Figure 4C).

### 3.4 KDM1A inhibition and BMP‐2 stimulation attenuated neointimal hyperplasia and affected VSMC behaviour in vivo

**Figure 5:**
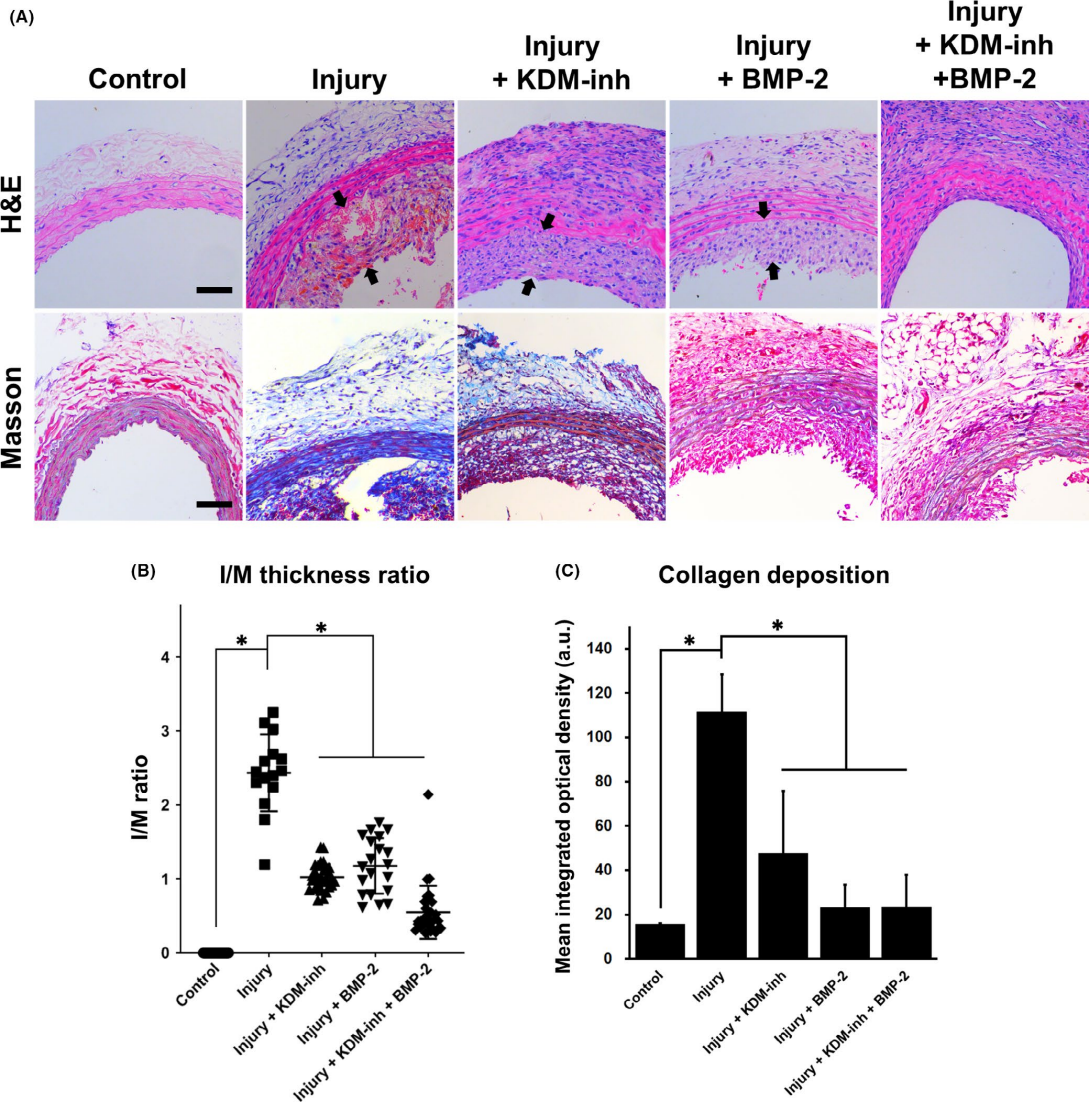
Histological assessment of neointimal hyperplasia and collagen deposition in aortic tissues. (A) H&E staining reveals the extent of neointimal formation (indicated between black arrows) after experimental rats were subjected to aortic endothelial balloon injury and/or administration of KDM‐inh and/or recombinant BMP‐2. Masson's trichrome staining indicates areas of fibrosis and collagen deposition in deep blue. Scale bar, 100 μm. (B) Quantification of neointimal formation via ratio of intimal to medial (I/M) thickness. ImageJ measurements of intimal and medial thickness were taken at 5‐10 equidistant segments from each section, using three H&E sections from each group. The I/M ratio was calculated as the average of all measurements and presented as the mean ± SD, *P < .05. (C) Quantification of collagen deposition. Image‐Pro Plus was used to measure the mean integrated optical density of collagen‐positive (blue) areas on each section. The data are presented as the mean ± SD of three independent replicates, *P < .05

Histological examination of aortic endothelial tissues (Figure 5) revealed a remarkable increase in the intimal thickness in rats subjected to aortic endothelial balloon injury compared to that in control rats, indicating the onset of neointimal hyperplasia. The administration of KDM‐inh and BMP‐2 individually reduced the neointimal thickness to a certain extent, but their combination prevented neointimal formation almost completely. The ratio of intimal to medial (I/M) thickness was also measured as an indication of neointimal formation (Figure 5B). In terms of collagen deposition, Masson's trichrome staining (Figure 5A,C) showed significant areas of dark blue staining (collagen fibres) in balloon‐injured rats, covering almost the entire area of the tissue. With KDM‐inh treatment, the distribution and intensity of collagen fibres were greatly attenuated, and BMP‐2 seemed to exert an even greater effect. The combined treatment of KDM‐inh and BMP‐2 effectively eliminated collagen deposition and the occurrence of aortic fibrosis.

**Figure 6:**
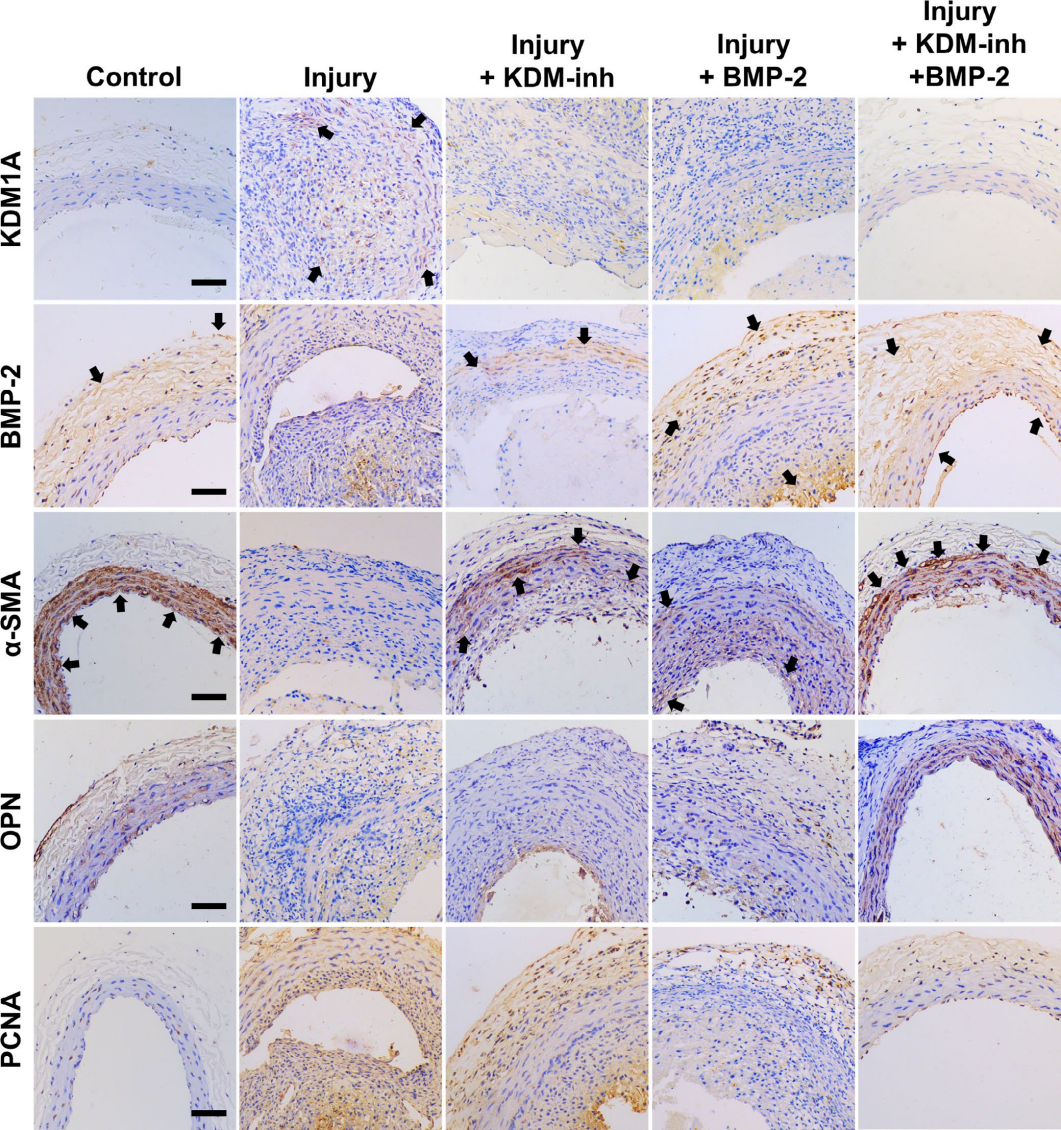
Immunohistochemical evaluation of KDM1A and BMP‐2 expression and VSMC phenotype and proliferation in aortic tissues. Tissue samples were staining for KDM1A, BMP‐2, α‐SMA, OPN, or PCNA after experimental rats were subjected to aortic endothelial balloon injury and/or administration of KDM‐inh and/or recombinant BMP‐2. Positive staining for KDM1A, BMP‐2, α‐SMA, OPN, or PCNA is shown in light brown and pointed out by black arrows. Each image is a representative of the respective group. Scale bar, 100 μm

Because we aimed to elucidate the relationship between KDM1A and BMP‐2 in vascular injury, we examined the expression of these proteins by means of immunohistochemistry in balloon‐injured rats treated with KDM‐inh and/or recombinant BMP‐2 (Figure 6). Consistent with the in vitro results of VSMCs reported in Section 3.1, aortic endothelial balloon injury led to an increase in the expression of KDM1A and a decrease in that of BMP‐2. The individual addition of KDM‐inh and BMP‐2 slightly reduced the expression of KDM1A and enhanced that of BMP‐2. The combined effect of the two was greater than that of individual administration, resulting in the absence of KDM1A and high distribution of BMP‐2 within the tissues.

Next, we investigated whether balloon‐induced vascular injury affected the phenotype and proliferation of VSMCs within the tissues. In rats subjected to aortic endothelial balloon injury, the expression of the contractile VSMC marker α‐SMA showed a drastic decrease, whereas that of the synthetic marker OPN was elevated. The administration of KDM‐inh and BMP‐2 separately upregulated the expression of α‐SMA and downregulated that of OPN. As VSMC phenotype is directly related to cell proliferation (which in turn correlates with neointimal formation), immunohistochemistry was performed for PCNA as an indication of VSMC proliferation. We observed significant areas of positive PCNA staining in tissues subjected to balloon injury, which is consistent with the results of H&E staining that suggested the onset of neointimal formation. The application of KDM‐inh effectively inhibited VSMC proliferation to a small extent, whereas BMP‐2 exerted a much larger inhibitory effect. Unsurprisingly, the combined administration of KDM‐inh and BMP‐2 suppressed VSMC proliferation to a much greater extent than did the individual components.

### 3.5 Regulation of neointimal hyperplasia via BMP‐2 involves SMAD and BMPR signalling

**Figure 7:**
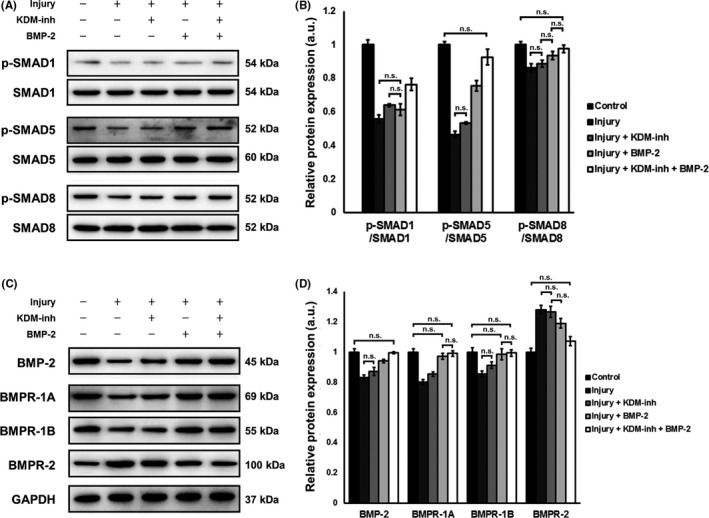
Involvement of SMAD and BMPR signalling pathways in KDM1A/BMP‐2‐mediated regulation of neointimal hyperplasia. (A) Western blot detection and (B) quantification of the relative protein expression of p‐SMAD/SMAD (1, 5, and 8) in tissue samples after experimental rats were subjected to aortic endothelial balloon injury and/or administration of KDM‐inh and/or recombinant BMP‐2. (C) Western blot detection and (D) quantification of the relative protein expression of BMP‐2, BMPR‐1A, BMPR‐1B, and BMPR‐2 (all normalized to GAPDH) in tissue samples after experimental rats were subjected to aortic endothelial balloon injury and/or administration of KDM‐inh and/or recombinant BMP‐2. For (A) and (C), portions of full western blot membrane images were cropped to show relevant bands. The data are presented as the mean ± SD of three independent replicates, n.s., not significant at *P* < .05 (comparisons between unmarked groups are all significant at *P* < .05)

With regard to BMP‐2 signalling, we were interested in the involvement of SMADs during neointimal formation. We proceeded to evaluate the expression of various SMADs in response to aortic endothelial balloon injury (Figure 7A,B). In particular, SMADs 1, 5, and 8 have been widely implicated in BMP‐2 signalling. Herein, we noticed that balloon‐induced injury led to a drastic decrease in the phosphorylation of all three SMADs (*P* < .05), whereas KDM‐inh and BMP‐2 individually recovered their expression to various degrees (not all differences were significant). However, the combination of KDM‐inh and BMP‐2 effectively triggered the phosphorylation of SMADs 1, 5, and 8 after injury (*P* < .05), and in the case of SMAD5 and SMAD8, phosphorylation was restored to levels close to those of control rats that were not subjected to injury.

Knowing that downstream BMP/BMPR signalling is critically implied in SMAD activity, we specifically investigated the behaviour of BMP‐2 itself and receptors 1A, 1B, and 2 after aortic endothelial balloon injury was carried out in rats. The results of western blot (Figure 7C,D) illustrated that balloon‐induced injury suppressed the expression of BMP‐2, BMPR‐1A, and BMPR‐1B, but elevated that of BMPR‐2 compared to those in control rats (*P* < .05). KDM‐inh and BMP‐2 separately elevated the expression of BMP‐2, BMPR‐1A, and BMPR‐1B after balloon injury, although to various extents (again, not all differences were significant). However, in all cases, the combined administration of the two caused significant increases in BMP‐2, BMPR‐1A, and BMPR‐1B expression after balloon injury (*P* < .05). The opposite was observed for BMPR‐2, whereby rats treated with both KDM‐inh and BMP‐2 exhibited a large decline in BMPR‐2 expression compared to that in non‐treated injured rats (*P* < .05).

## 4 DISCUSSION

The transition from a contractile to synthetic phenotype is a necessary step in the early developmental stages of vascular diseases such as atherosclerosis and restenosis.^34^ Aside from excessive proliferation, this transition is typically characterized by the onset of inflammation and neointimal formation.^6^, ^35^, ^36^, ^37^, ^38^ Consequently, the prevention or reversion of this process can aid in the control of vascular disorders. The findings of our in vitro study collectively showed that the inhibition of KDM1A expression significantly downplayed the effect of Ang‐II in the induction of contractile‐to‐synthetic phenotypic modulation in VSMCs (Figure 3A,B). This is complemented by the observation that KDM‐inh diminished the Ang‐II–triggered production of factors associated with inflammation (IL‐6, IL‐18, ICAM‐1, and MCP‐1), cell proliferation (PDGF and FGF‐2) and extracellular matrix remodelling (MMP‐2) (Figure 3E). Thus, we suggest that KDM1A is required for Ang‐II to function properly in inducing the contractile‐to‐synthetic transition of VSMCs. Simultaneously, we induced arterial wall thickening in a rat model using the classic method of balloon catheter injury. Our in vivo results revealed that KDM1A deficiency, along with recombinant BMP‐2 treatment, contributed to the suppression of neointimal formation after aortic endothelial balloon injury (Figures 5 and 6). The therapeutic application of KDM1A, however, remains to be fully explored and validated.

A thorough understanding of the precise relationship between KDM1A and BMP‐2 will allow us to develop effective strategies for targeted local delivery of KDM1A‐based agents with therapeutic potential against neointimal hyperplasia and other vascular disorders. BMP‐2 is a widely studied and critical protein that is involved in a variety of biological phenomena, and its function may differ in osteogenic signalling, tumorigenesis, and vascular remodelling.^39^ In some cases, such as vascular calcification and atherosclerosis, BMP‐2 may have dual effects at various developmental phases.^40^ Meanwhile, though research on the involvement of KDM1A in vascular modulation is scarce in the literature, the interaction between KDM1A and BMP‐2 has been reported in several studies. In glioblastoma, KDM1A was stabilized via phosphorylation, which was triggered by glycogen synthase kinase 3β. This led to the demethylation of histone H3K4, in turn inhibiting the transcription of the *BMP‐2* gene and promoting glioblastoma tumorigenesis.^41^, ^42^ In a study of osteoblastic differentiation, KDM1A deficiency enhanced BMP‐2 signalling in human mesenchymal stem cells and mice and promoted an osteoblastic phenotype.^43^

It is reasonable to speculate that the above‐mentioned negative regulatory effects between KDM1A and BMP‐2 are also present in the vascular microenvironment. In other words, inhibiting the expression of KDM1A may achieve the effect of upregulating BMP‐2 expression, effectively activating its functions. The role of BMP‐2 has been well established in bone tissue engineering, and likewise, its implications in vascular remodelling and diseases are non‐trivial. In a well‐established study using rat aortic VSMCs, Nakaoka et al suggested that BMP‐2 inhibited neointimal hyperplasia caused by balloon injury, implicating the therapeutic potential of BMP‐2 in the prevention of vascular proliferative diseases.^23^ Our histological analysis of rat aortic tissues showed that KDM‐inh and BMP‐2 were able to attenuate neointimal formation and tissue fibrosis after balloon‐induced injury (Figure 5). We note also that BMP‐2 (and consequently, KDM‐inh) promoted the contractile phenotype in VSMCs and inhibited their proliferation, as signified by the increased expression of α‐SMA and decreased expression of PCNA in injured aortic tissues treated by BMP‐2 (Figure 6). This is complementary to our in vitro observations (Figures 1, 2, 3) and is consistent with other reports demonstrating the importance of BMP‐2 in the maintenance of contractile markers and suppression of proliferation in VSMCs.^40^, ^44^, ^45^

BMP‐2 often interacts with BMPRs and downstream SMADs, consequently resulting in a series of signal cascades.^46^ Whether BMP‐2 signalling is transduced via canonical or non‐canonical routes in vascular remodelling may depend on other components involved in the signalling cascade. For example, BMP signalling induced nuclear recruitment of myocardin‐related transcription factors (MRTFs) to an α‐SMA promoter and modulated VSMC phenotype. This interaction between BMPs and MRTFs was possibly due to non‐SMAD pathways.^40^ Herein, we revealed that KDM‐inh suppressed neointimal hyperplasia in injured aortic tissues by mediating canonical SMAD‐related pathways (Figure 7A,B). The same phenomenon was observed when injured tissues were treated by BMP‐2 (Figure 5). The activation of R‐SMADs (1, 5, and 8) upon administration of KDM‐inh and BMP‐2 was accompanied by enhanced expression of BMPR‐1A and BMPR‐1B, but BMPR‐2 signalling was disrupted (Figure 7C,D).

The premise and results of our investigation may seem to disagree with a number of studies reporting that BMP‐2 contributes to vascular calcification, and thus atherosclerosis. We propose several explanations for the controversy. First, KDM1A signalling, which is the key to this study, could be much more potent than BMP‐2 signalling. KDM1A itself may have unknown, unreported pro‐inflammatory or pro‐atherogenic effects, which may override those of BMP‐2. While KDM1A targets BMP‐2 by decreasing its expression, if the effect of KDM1A is potent enough in inducing the synthetic phenotype of VSMCs or neointimal hyperplasia, then the function of BMP‐2 becomes passive. In other words, the downregulation of BMP‐2 is merely a result of KDM1A targeting and would itself have negligible effects on VSMC behaviour and vascular calcification. In the same manner, the inhibition of KDM1A signalling by KDM‐inh results in an upregulation of BMP‐2, but this upregulation would have little impact on vascular remodelling, as the effect of KDM1A inhibition is far greater. In fact, KDM1A may interfere with BMP signalling by impairing or altering the action of BMPR‐2 (Figure 7C,D), which is normally downregulated in pro‐atherogenic conditions.^47^ Another possibility is that, as we have mentioned previously, BMP‐2 signalling plays pleiotropic roles in regulating cellular processes. Depending on the specific growth conditions and state of proliferation, BMP‐2 may have different effects on VSMCs in vitro or function as part of a continuum.^48^ It was also suggested that while BMP‐2 halts VSMC proliferation and causes cell cycle arrest, only further and continuous exposure to BMP‐2 will result in vascular calcification.^48^ Because of the prominent implications of BMP‐2 in osteochondrogenic functions, in future studies, it will be interesting to explore whether these functions have an influence on VSMC phenotype and vascular remodelling in the context of neointimal hyperplasia. Specific molecular interactions between KDM1A and various BMPRs, especially BMPR‐2, will also be investigated to provide further insights into the mechanism of the KDM1A/BMP signalling axis.

Collectively, the findings reported here demonstrated the regulatory correlation between KDM1A and BMP‐2 signalling in vascular remodelling, specifically in terms of VSMC phenotypic modulation (in vitro and in vivo) and the control of neointimal hyperplasia. We speculate that an in‐depth understanding of the functions and implications of KDM1A will offer insights into the development of innovative strategies for the treatment of vascular disorders.

## CONFLICT OF INTEREST

The authors declare that there is no conflict of interest.

## AUTHORS' CONTRIBUTIONS

XZ and HY designed the study; XZ, TH, and HZ performed cell culture and Western blot; XZ, TH, and WP carried out immunohistochemistry and microscopic experiments; XZ, TH, YZ, and QL were involved in in vivo experiments; XZ and TH performed flow cytometry; XZ, TH, WP, YZ, and QL analysed the data; XZ, TH, and HY drafted and revised the manuscript. All authors read and approved the final manuscript.

## Data Availability

The data that support the findings of this study are available from the corresponding author upon reasonable request.
