# Supplementary material for: Inhibition of lysine‐specific demethylase 1A suppresses neointimal hyperplasia by targeting bone morphogenetic protein 2 and mediating vascular smooth muscle cell phenotype
Source: Cell Prolif. 2019 Nov 18;53(1):e12711. doi: 10.1111/cpr.12711 (PMC6985674; doi:10.1111/cpr.12711)
Supplement: Supplementary file 1 [file CPR-53-e12711-s001.docx]

**SUPPLEMENTARY MATERIALS**

**Inhibition of lysine-specific demethylase 1A suppresses neointimal hyperplasia by targeting bone morphogenetic protein-2 and mediating vascular smooth muscle cell phenotype**

**Running title:** KDM1A alters VSMC phenotype via BMP-2

Xiaobo Zhang^1^, Heng Zhai^2^, Wenpeng Peng^1^, Yong Zhou^3^, Qi Li^3^, Tao Huang^3^, Haifeng Yang^3,*^

^1^Department of Cardiology, Union Hospital, Tongji Medical College, Huazhong University of Science and Technology, Wuhan, China

^2^Department of Neurology, Union Hospital, Tongji Medical College, Huazhong University of Science and Technology, Wuhan, China

^3^Department of Neurosurgery, Union Hospital, Tongji Medical College, Huazhong University of Science and Technology, Wuhan, China

***Corresponding author**

Haifeng Yang

Department of Neurosurgery, Union Hospital

Tongji Medical College, Huazhong University of Science and Technology

No. 1277 Jiefang Avenue, Wuhan, Hubei, 430022, China

Tel: (+86) 18627923316; Email: yanghaifeng_1984@163.com

**DETAILED MATERIALS AND METHODS**

***VSMC isolation and culture***

VSMCs were isolated from Sprague Dawley rats (obtained from the Hubei Disease Prevention and Control Center, Wuhan, China) in an aseptic environment. The rats were anesthetized with 30 mg/kg pentobarbital. The middle section of thoracic and abdominal aortic tissues was extracted from the rats and rinsed three times with phosphate-buffered saline (PBS, PAB180003, Bio-swamp, Wuhan, China) containing penicillin-streptomycin (PAB180086, Bio-swamp). The tissues were then cultured in 2 mL of high-glucose Dulbecco's modified Eagle medium (SH30022.01, South Logan, UT) and cut into pieces of approximately 1 mm^3^. After incubation in a polylysine-coated T25 flask at 37°C for 6 h in an atmosphere containing 5% CO_2_, the tissues were observed to adhere to the wall of the flask. The medium was thereafter replaced with smooth muscle cell medium (SMCM, 1101, Sciencell Research Laboratories, Carlsbad, CA) and changed every three days. After 4–6 days, VSMCs were seen to emerge from the tissues, and 80–90% confluence was reached after approximately two weeks.

***VSMC stimulation with angiotensin-II (Ang-II) and KDM1A inhibitor***

The in vitro model of phenotypical switching was constructed using Ang-II as a stimulus to trigger VSMC proliferation and transformation from a contractile to a synthetic state. VSMCs were cultured until they reached 80–90% confluence and incubated with 10^-7^ mol/L Ang-II (A107852, Aladdin, Shanghai, China) for 24 h. To investigate the influence of KDM1A in the determination of VSMC phenotype, the cells were pre-treated for 2 h with 10^-7^ mol/L ORY-1001 (S7795, Selleck Chemicals, Houston, TX), an inhibitor of KDM1A (KDM-inh), before incubation with Ang-II.

***3-(4,5-dimethylthiazol-2-yl)-2,5-diphenyltetrazolium bromide (MTT) assay***

Cell in the logarithmic growth phase were seeded in a 96-well plate at 5 × 10^3^ cells per well with 180 μL of medium. The plate was incubated overnight at 37°C in an atmosphere containing 5% CO2 and treated for as outlined in section 1.2. MTT reagent (5 mg/mL, 20 μL per well, PAB180013, Bio-swamp) was added and the adherent cells were further incubated for 4 h. Thereafter, the medium was removed and 150 μL of dimethyl sulfoxide was added to each well. The plate was shaken for 10 min and the absorbance of each well was measured using a plate reader (Multiskan FC, Thermo Fisher Scientific, Waltham, MA, USA) at 490 nm.

***Scratch assay***

VSMCs were seeded in a 6-well plate at 1 × 10^6^ cells per well and treated as described in section 1.2. The cells were allowed to grow until they covered the bottom of the well in a monolayer. A pipette tip was positioned perpendicular to the cell monolayer and a scratch was made quickly in the monolayer. Thereafter, the well was washed with PBS to remove the detached cells, and serum-free medium was added to the well. Images of the scratch were taken using an optical microscope immediately after scratching (0 h) and 24 h after incubation at 37°C in an atmosphere containing 5% CO_2_.

***EdU staining for cell proliferation***

VSMC proliferation was assessed using an EdU staining kit (PAB180087, Bio-swamp). VSMCs were seeded in a 96-well plate and treated as described in section 2.2. EdU reagent (solution A, included in the kit) was added to SMCM at a ratio of 1:1000 to prepare a working solution of EdU at a final concentration of 50 μM. Then, 100 μL of the working solution was added to each well and the cells were further incubated for 2 h at 37°C in an atmosphere containing 5% CO_2_. The solution was then removed and the cells were washed twice with PBS for 5 min each to remove residual EdU. Cells were fixed with 100 μL of 4% paraformaldehyde in PBS per well for 30 min, after which the solution was removed and 2 mg/mL glycine was added. The plate was shaken for 5 min and the cells were washed with PBS for 5 min. For permeabilization, 0.5% Triton X-100 in PBS was added at 100 μL per well and the plate was incubated for 10 min. The cells were washed with PBS for 5 min, and 100 μL of 1× Apollo® staining reagent (provided in the kit) was added to each well. The plate was incubated at room temperature in the dark for 30 min. The staining reagent was removed, and the cells were permeabilized again with 0.5% Triton-X in PBS three times for 10 min each. Next, the cells were washed twice with 100 μL of methanol for 5 min each and once with PBS for 5 min. Hoechst33258 reagent (provided in the kit) was diluted in deionized water at 1:100 to prepare a working solution and stored in the absence of light. Then, the cells were incubated with Hoechst33528 at 100 μL per well for 30 min at room temperature in the dark. The staining solution was removed and the wells were washed three times with PBS, and the cells were observed using a fluorescence microscope (DMIL LED, Leica Microsystems, Wetzlar, Germany).

***Transwell migration assay***

For the Transwell assay, a 24-well plate was coated with 80 µL of Matrigel (354230, Corning Inc., Corning, NY) and incubated at 37°C for 1 h. VMSCs were resuspended at 1 × 10^5^ cells per mL in serum-free SMCM and seeded in the upper Transwell chambers at 0.5 mL cells per well. The bottom wells were filled with 0.75 mL of 10% fetal bovine serum (FBS). The seeded VSMCs were treated as described in section 1.2 and the plate was incubated at 37°C for 48 h. The medium was removed and 1 mL of 4% formaldehyde was added to each well for 10 min for fixation. After removal of the fixative solution, VSMCs that migrated to the bottom chamber were stained with 1 mL of 0.5% crystal violet solution (PAB180004, Bio-swamp) in each well for 30 min. The cells were washed three times with PBS and dried. Cell count was obtained using an optical microscope.

***Flow cytometry***

For the analysis of cell cycle progression, the cells were centrifuged at 1000 × g for 5 min and the supernatant was discarded. The cells were resuspended in 300 μL of PBS containing 10% FBS and 700 μL of anhydrous ethanol and fixed at -20°C for at least 24 h. The fixed samples were centrifuged at 3000 × g for 30 s and the supernatant was removed. The cells were washed twice with 1 mL of pre-cooled PBS and resuspended in 1 mg/mL RNAse A at 37°C to digest cellular RNA. Thereafter, the cells were labeled with 400 μL of 50 μg/mL propidium iodide in the dark for 10 min, and the proportion of cells in each phase of the cell cycle was determined by flow cytometry. For the analysis of apoptosis, approximately 1 × 10^6^ cells were resuspended in PBS and centrifuged at 1000 × g for 5 min at 4°C. After the supernatant was discarded, the cells were mixed with 1 mL of pre-cooled PBS and centrifuged at 1000 × g for 5 min at 4°C. The supernatant was removed and the previous step was repeated. Then, 200 μL of binding buffer was added to the cells, after which 10 μL of annexin V-fluorescein isothiocyanate and 10 μL of propidium iodide were added. The cell suspension was gently mixed and incubated for 30 min at 4°C in the dark, and 300 μL of binding buffer was added. All flow cytometry experiments were performed using a NovoCyte apparatus (ACEA Biosciences, Inc., San Diego, CA, USA) The data were analyzed using NovoExpress software (ACEA Biosciences, Inc.)

***Immunofluorescence***

VSMCs were treated as described, resuspended, and seeded onto coverslips placed in the wells of a well plate. The cells were washed twice with PBS and fixed with 4% paraformaldehyde at room temperature for 30 min. After three washes with PBS for 3 min each, the cells were permeabilized with 0.5% Triton X-100 in PBS at room temperature for 20 min and washed again three times with PBS for 3 min each. The cells were blocked with 5% bovine serum albumin for 1 h at 37°C, after which they were incubated with rabbit primary antibodies against α-SMA (1:100, ab5694, Abcam, Cambridge, UK) or OPN (1:250, ab75285, Abcam) at 4°C overnight. The samples were washed three times with PBS for 5 min each and incubated with Alexa Fluor 488-conjugated Affinipure goat anti-rabbit IgG(H+L) secondary antibodies (1:200, PAB160027, Bio-swamp) for 1 h at 37°C in the dark, followed by three washes with PBS for 5 min each. The coverslips were removed from the well plate and mounted on microscope slides using sealing solution containing 4’,6-diamidino-2-phenylindole for nuclear staining. The samples were observed using a fluorescence microscope (Eclipse TS100-F, Nikon, Tokyo, Japan).

***Enzyme-linked immunosorbent assay (ELISA)***

ELISA was performed to quantify the production of growth factors and inflammatory cytokines from VSMCs stimulated with Ang-II and/or KDM-inh. ELISA kits (all from Bio-swamp) for MMP-2 (RA20502), ICAM-1 (RA20614), PDGF (RA20454), FGF-2 (RA20438), IL-6 (RA20607), IL-18 (RA20058), MCP-1 (RA20492), and TGF-β (RA20080) were used based on the manufacturer’s instructions.

***In vivo model establishment***

All animal experiments were performed in accordance to the Guidelines for Animal Care and Use of the Model Animal Research Institute at Wuhan Myhalic Biotechnology Co., Ltd. and were approved by the institutional review board (approval number: HLK-20180925-01). Fifty male Sprague Dawley rats (8 weeks old, 250–280 g, purchased from the Animal Experiment Center of China Three Gorges University) were randomly divided into five groups (n = 10 per group): sham operation (Control), aortic endothelial balloon injury (Injury), KDM-inh treatment after injury (Injury + KDM-inh), recombinant BMP-2 administration after injury (Injury + BMP-2), and treatment with both KDM-inh and recombinant BMP-2 after injury (Injury + KDM-inh + BMP-2). Before the experiment, the rats were anesthetized with 40 mg/kg sodium pentobarbital (P3761, Sigma-Aldrich, St. Louis, MO). Sham operation was performed via surgical dissection of the common carotid artery on rats, after which the wound was sutured without any treatment. For the establishment of the aortic endothelial balloon injury model, a custom-made 2F balloon catheter was inserted into the left common carotid artery of the rats and delivered to the lower end of the abdominal aorta. The balloon was filled with 0.1 mL of physiological saline, and the catheter was pulled to remove the arterial endothelium, completing the induction of endothelial injury. For KDM-inh and BMP-2 treatment, injured rats were intraperitoneally injected daily with 2 mL of 10 μM ORY-1001 (an inhibitor of KDM1A) or 100 ng/mL recombinant BMP-2 protein (120-02, Peprotech, Rocky Hill, NJ), respectively. Combined treatment was performed by injecting a mixture of 1 mL of ORY-1001 and 1 mL of recombinant BMP-2. Sham-operated rats and those subjected to balloon injury only were injected daily with 2 mL of physiological saline. Aortic tissues collected from the rats 7, 14, or 28 days after the operation were fixed and stored at -80°C.

***Quantitative reverse transcription polymerase chain reaction (qRT-PCR)***

RNA was extracted using TRIzol (15596026, Ambion, Inc., Foster City, CA, USA) and reverse-transcribed into cDNA using the RevertAid First Strand cDNA Synthesis Kit (K1622, Thermo Scientific) and TaqMan microRNA assay kit (Applied Biosystems, Foster City, CA, USA). qRT-PCR was performed using the SYBR Green PCR kit (KM4101, KAPA Biosystems, Wilmington, MA) with the following primer sequences: KDM1A forward, 5'-GCTCCTATTCTTATGTGG-3' and reverse, 5'-AGTTGCGGATTGTATG-3'; BMP-2 forward, 5'-GCGAGTTTGAGTTGAGG-3' and reverse, 5'-TGAGCACGGTGTTGG-3'; GAPDH forward, 5'-CAAGTTCAACGGCACAG-3' and reverse, 5'-CCAGTAGACTCCACGACAT-3'. The experimental conditions were as follows: initial denaturation at 95°C for 3 min; 39 cycles of denaturation at 95°C for 5 s, annealing at 56°C for 10 s, and extension at 72°C for 25 s; and final extension at 65°C for 5 s and 95°C for 50 s. Data acquisition was carried out using the QuantStudio™ 6 Flex Real-Time PCR System (Applied Biosystems) and analyzed with the 2^-ΔΔCt^ method.

***Western blot***

Proteins extracted from cell or tissue samples were subjected to western blot. For in vitro experiments, the cells were washed twice with cold PBS and lysed with radioimmunoprecipitation assay (RIPA) buffer (PAB180006, Bio-swamp) at 4°C. The lysed cells were heated for 10 min at 95°C and centrifuged at 12000 × g for 10 min, and the supernatant was stored at -80°C. For in vivo experiments, approximately 20 mg of tissue was treated with 150–250 μL of RIPA buffer. The tissues were homogenized completely and centrifuged at 12000 × g for 15 min. The supernatant was then stored at -80°C until use. The proteins in the supernatant of cell and tissue samples were quantified using a bicinchoninic acid assay kit (PAB180007, Bio-swamp) according to the manufacturer’s protocols. For sodium dodecyl sulfate-polyacrylamide gel electrophoresis, 20 μg of each protein sample was loaded into the gel. The proteins were then transferred to polyvinylidene fluoride membranes (IPVH00010, Millipore, Burlington, MA, USA) and blocked with 5% non-fat skim milk in PBS/Tween 20 (PBST) for 2 h at room temperature. Next, the membranes were incubated overnight at 4°C with rabbit primary antibodies (all obtained from Bio-swamp, diluted at 1:1000) against KDM1A (PAB30728), BMP-2 (PAB30060), α-SMA (PAB35544), OPN (PAB42433), BMPR-1A (PAB33013), BMPR-1B (PAB40430), BMPR-2 (PAB34155), SMAD1 (PAB30594), p-SMAD1 (PAB43293-P), SMAD5 (PAB38625), p-SMAD5 (MAB43553-P), SMAD8 (PAB35326), p-SMAD8 (PAB43637-P), eNOS (PAB32306), or GAPDH (PAB36264). After the membranes were washed three times with PBST for 5 min each, they were incubated with goat anti-rabbit IgG secondary antibodies (1:20000, PAB160011) for 1 h at room temperature, then washed again three times with PBST for 5 min each. An enhanced chemiluminescent reagent (WBKLS0010, Millipore) was applied for immunodetection, and the protein bands were visualized using a Tanon-5200 system (Tanon, Shanghai, China) and analyzed using Tanon software.

***Hematoxylin/eosin (H&E) and Masson’s trichrome staining***

Extracted aortic tissues were cut into small pieces (1.5 cm × 1.5 cm × 0.3 cm) and fixed in 10% formalin for 48 h. The fixed specimens were then washed with water and dehydrated using an automatic tissue dehydration apparatus (TKD-TSF, Hubei KangQiang Medical Devices Co., Ltd., Xiaogan, China) for 8 h. Next, the specimens were embedded in melted paraffin wax, and the paraffin-embedded tissue blocks were cooled for 30 min. The embedded tissues were cut with a rotary microtome (RM2235, Leica Biosystems, Buffalo Grove, IL, USA) into sections with a thickness of approximately 3–5 μm. For H&E staining, tissue sections were deparaffinized and rinsed in water for 2 min. Then nuclear staining was performed using hematoxylin (PAB180015, Bio-swamp) for approximately 5 min and washed in running water for 2 min to remove the dye. After being immersed briefly in 1% hydrochloric acid and bluing, the cytoplasm was stained with 0.5% eosin (PAB180016, Bio-swamp) for 3 min and the tissues were washed with distilled water. The tissues were finally immersed in 80% ethanol for 30 s, 95% ethanol for 30 s, 100% ethanol for 3 min, xylene I for 5 min, and xylene II for 5 min and sealed with neutral balsam mounting medium (PAB180017, Bio-swamp). Masson’s trichrome staining was performed using an assay kit (PAB180023, Bio-swamp). All reagents were provided in the kit and prepared as described in the protocols. Stained tissues were viewed under a microscope (MD1000, Leica, Solms, Germany) and analyzed using Leica Application Suite software (Leica Microsystems).

***Quantification of neointimal formation and collagen deposition***

Neointimal formation was quantified by measuring the ratio of intimal-to-medial (I/M) thickness in aortic tissues using ImageJ. Measurements of intimal and medial thickness were taken at 5–10 equidistant segments from each section, using three H&E sections from each group. The I/M ratio was calculated as the average of all measurements. For the quantification of collagen deposition, Image-Pro Plus was used to measure the mean integrated optical density of collagen-positive (blue) areas on each Masson-stained section.

***Immunohistochemical staining***

Tissue sections were prepared as described in section 1.13. Prior to staining, the sections were heated at 65°C for 1 h and soaked twice in toluene for 15 min. Deparaffinized sections were immersed in ethanol in a graded series of concentrations (100%, 95%, 85%, 75%) for 5 min at each concentration, then rinsed in running tap water for 10 min. For antigen retrieval, the sections were incubated with 0.01 M sodium citrate buffer for 15 min. After three washes in PBS for 3 min each, the tissues were incubated with 3% H_2_O_2_ for 10 min in a humid container to removal endogenous peroxidase. Blocking was performed using 0.5% bovine serum albumin for 30 min, after which the sections were incubated at 4°C overnight with rabbit primary antibodies against KDM1A (1:50, PAB30728, Bio-swamp), BMP-2 (1:100, PAB30060, Bio-swamp), α-SMA (1:50, PAB35136, Bio-swamp), OPN (1:100, ab8448, Abcam), and PCNA (1:100, PAB30083, Bio-swamp). After three washes with PBS for 5 min each, the sections were incubated with MaxVision™ horseradish peroxidase-conjugated anti-rabbit secondary antibodies (1:200, PAB160022, Bio-swamp) for 30 min at room temperature and washed against three times with PBS for 3 min each. Diaminobenzidine solution was then added to the sections and when color appeared, the sections were washed with running tap water to remove the dye. Nuclear staining was performed for 3 min using hematoxylin. After being soaked in ethanol (75%, 85%, 95%, and 100%; 5 min at each concentration), the tissues were sealed with neutral balsam mounting medium, imaged under a microscope, and analyzed using Leica Application Suite.

***Statistical analysis***

All experiments were performed in triplicates (n = 3) and the data are presented as the mean ± standard deviation (SD). Statistical analysis was carried out by one-way analysis of variance with Tukey’s test for multiple comparisons using OriginPro 8. ImageJ ver. 1.8 and Image-Pro Plus 6.0 were used for image analysis. p < 0.05 is considered statistically significant.


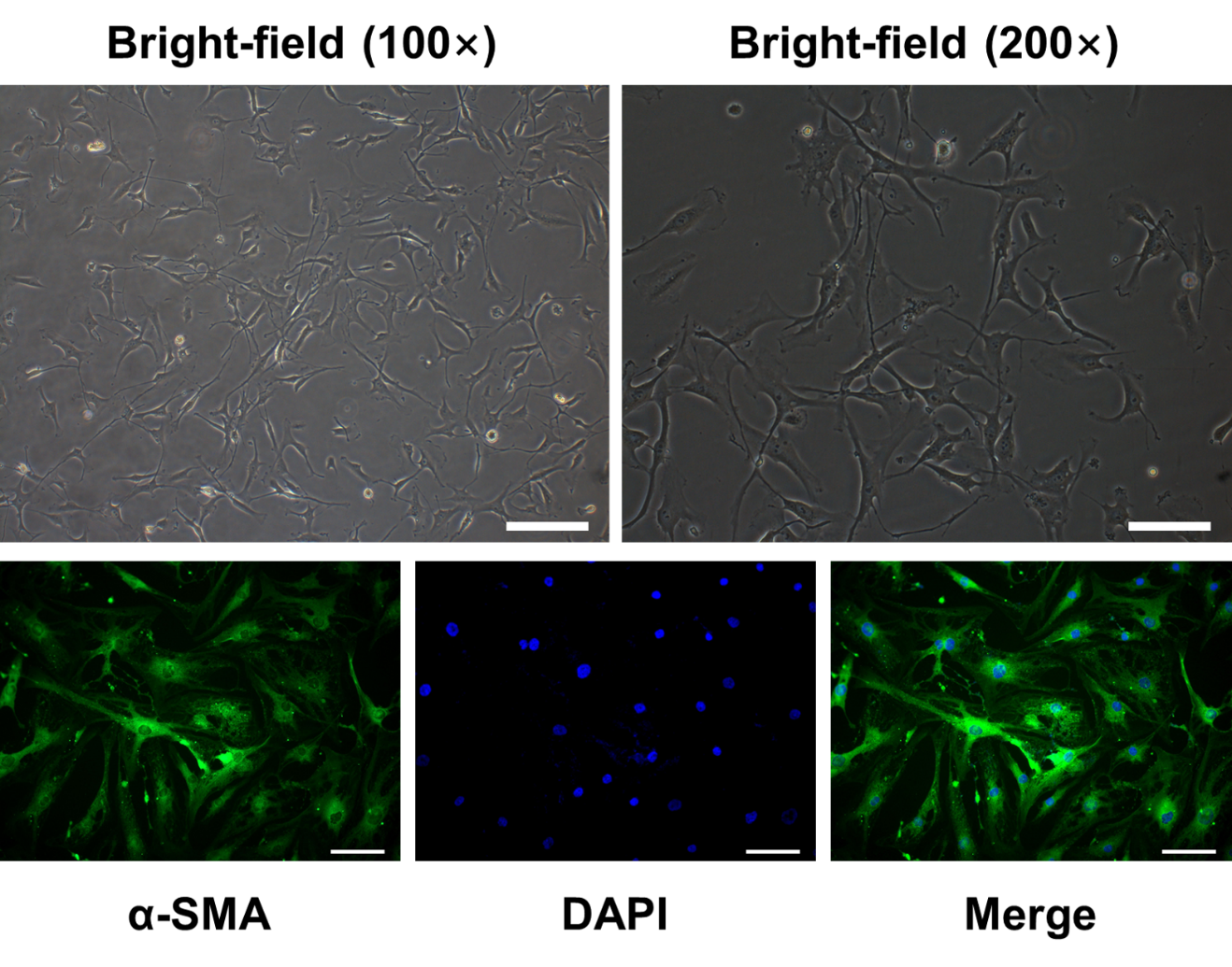


**Figure S1. Identification of isolated VSMCs using bright-field and fluorescence microscopy.** Typical peak-valley-like structures were observed by bright-field microscopy, signifying the successful isolation of VSMCs from Sprague Dawley rats. 100×, scale bar = 200 μm; 200×, scale bar = 100 μm. Fluorescence microscopic observation of the contractile VSMC marker α-SMA showed positive staining (green), confirming successful VSMC isolation. For fluorescence images, scale bar = 100 μm.


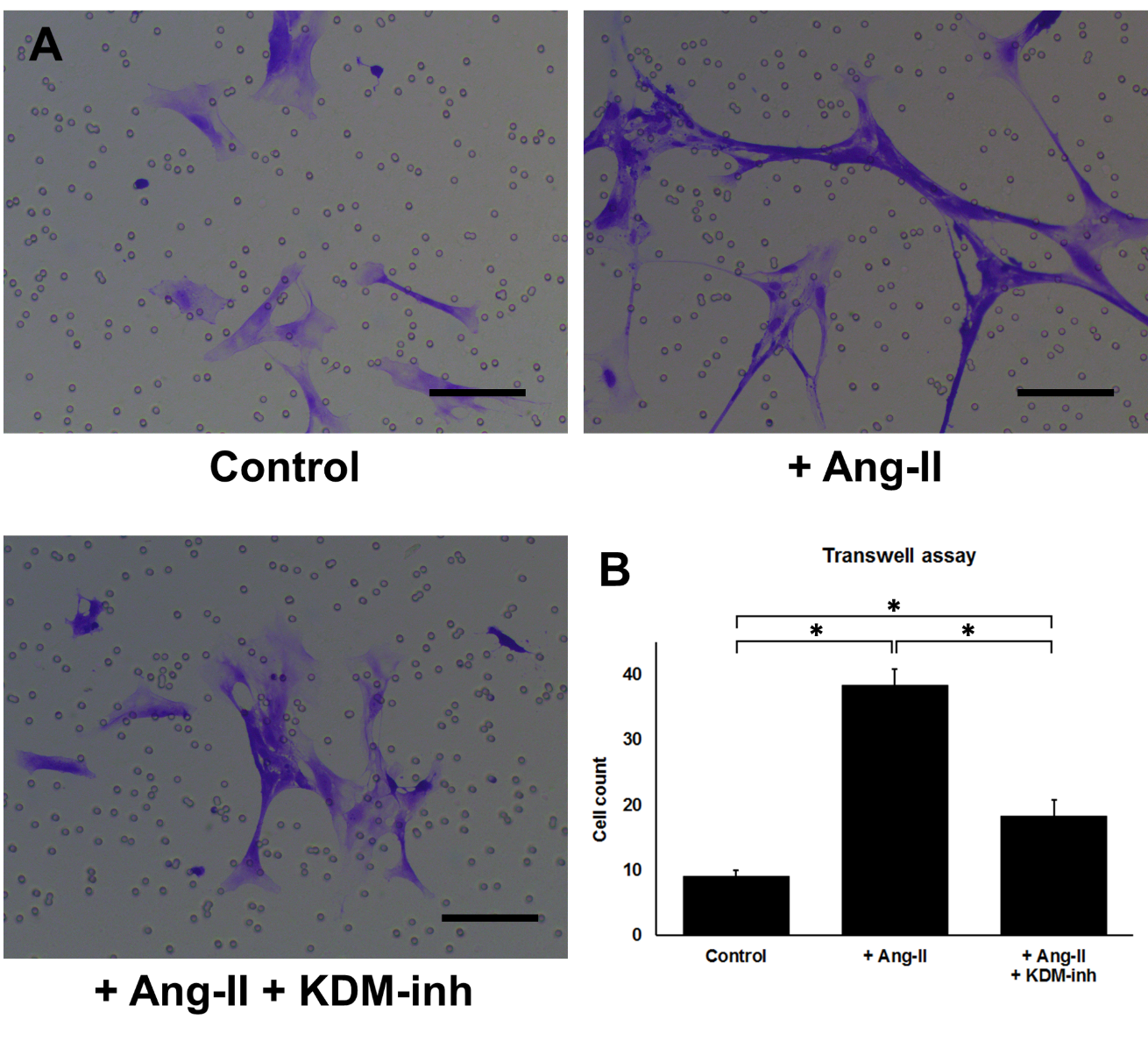


**Figure S2. Transwell assay of VSMC migration.** (A) Microscopic images of VSMCs subjected to crystal violet staining after Transwell assay. Ang-II promoted VSMC migration, whereas KDM-inh counteracted the effect of Ang-II. Scale bar = 100 μm. (B) Quantification of cell count. The data are presented as the mean ± SD of three independent replicates, *p < 0.05.
